# When fetal hydronephrosis is suspected antenatally—a qualitative study

**DOI:** 10.1186/s12884-015-0791-x

**Published:** 2015-12-22

**Authors:** Marie Oscarsson, Tomas Gottvall, Katarina Swahnberg

**Affiliations:** 1 Department of Health and Caring Sciences, Linnaeus University, Stagneliusgatan, 14, 391 82 Kalmar, Sweden; 2 Division of Obstetrics and Gynaecology, Department of Clinical and Experimental Medicine, Faculty of Health Sciences, Linköping University, 581 83 Linköping, Sweden; 3 Division of Gender and Medicine, Department of Clinical and Experimental Medicine, Faculty of Health Sciences, Linköping University, Linköping, Sweden

**Keywords:** Fetal hydronephrosis, Pregnancy, Ultrasound, Qualitative analysis

## Abstract

**Background:**

The information about fetal malformation findings during the ultrasound examination often comes unexpectedly, and the women and their partners may not necessarily receive any conclusive statement on the prognosis. A finding such as fetal hydronephrosis range from being a soft markers or mild anomaly, to a serious condition associated with neonatal morbidity and mortality. The aim of this study was to explore women’s reactions to the discovery of fetal hydronephrosis in the context of uncertainty regarding the prognosis.

**Methods:**

Ten women were interviewed and the interviews were conducted six to twelve months after the women gave birth. They had experience of suspected fetal hydronephrosis in gestational week 18–20. The interviews were recorded, transcribed verbatim and analysed using constant comparative analysis.

**Results:**

The core category, ‘Going through crisis by knowing that you are doing the right thing’ illustrates the meaning of women’s reactions and feelings. It illuminates the four categories: ‘*When the unexpected happens*’– on the one hand, women had positive views that the suspicious malformation could be discovered; however, on the other hand, women questioned the screening. *‘To live in suspense during pregnancy’ –* the suspicious malformation caused anxiety and was a stressful situation. *‘Difficulties in understanding information’ –* the women thought they had limited knowledge and had difficulties in understanding the information. ‘*Suppress feelings and hope for the best’ –* the women tried to postpone the problem and thought they should deal with it after delivery.

**Conclusions:**

Women are worried irrespective of suspicious or severe malformations, and in need of information and counselling tailored to their individual needs. Other sources of support could be: written information, links to reliable sources on the Internet and possibilities for ongoing follow-ups.

## Background

In Sweden, ultrasound screening in early pregnancy is offered to all pregnant women as part of the standard programme for prenatal care. Most often, routine ultrasound is performed between 18 and 20 weeks gestation by specially trained midwives [1]. In some obstetrics units, ultrasound is also offered at 11–14 and 32–33 weeks of gestation. The birth rate in Sweden is approximately 113,000 per year, and about 97 % of the pregnant women have at least one ultrasound examination. The clinical grounds for the examination are: determination of gestational age, placental localisation and identification of multiple pregnancies [2]. Women desire to undergo ultrasound screening for reasons such as: check for fetal malformations and to see that everything is normal [3]. In addition to these medical reasons, women have also expressed a wish to see the baby, get assurance of the pregnancy and to have a picture of the baby [3].

The detection of indications of fetal malformations can put healthcare professionals and parents in ethically difficult situations [4, 5]. There are spectrums of findings of malformation from susceptible, treatable, to risk for handicap to lethal. Each of these outcomes has psychological impact on the parents [6]. Information about fetal malformation findings from the ultrasound examination often comes unexpectedly; moreover, initially, the women and their partners may not be given any conclusive statement on the prognosis. This often means that women have to undergo further examinations such as amniocentesis, chorionic villus sampling or further ultrasound diagnosis. Some of these suspected fetal malformations, for example, hydronephrosis (HN) can progress during the pregnancy into more severe and even deleterious conditions, while others do not develop into any harmful conditions [7]. Continuing a pregnancy following a diagnosis of a non-lethal malformation is frequently stressful for parents [6, 8]. A stress that is not alleviated until after the birth with confirmation that neonatal intervention is either not necessary or that the condition is treatable [8].

Pregnant women’s lack of knowledge about the purpose of the ultrasound results in them not being prepared for adverse findings [8]. Although women in Sweden receive general information, both oral and written in relation to ultrasound screening, they have reported difficulties in absorbing all the information [9]. Results from an Irish study [10] showed that some women felt that they received information at a level exceeding their needs, which even increased their anxiety. While other women required a lot of information in order to cope with stressful situations [10]. When fetal malformations are discovered, sometimes decisions need to be made within short time when the pregnant women are in an emotional despair [11]. This means that women have to make decisions about future examinations or in worst cases make choices about terminations. In Sweden, abortion is permitted for pregnancies up to gestational week 18 if the woman requests. After this week, the National Board of Health and Welfare must approve a request for termination [12]. The National Board can approve request up to gestational week 22 in cases of severe indications, for example serious malformations of the fetus. Health professional’s support and recommendations therefore become crucial for the women’s decisions [9].

It is well-known that ultrasound screening during pregnancy has an impact on women’s well-being; thus, when a fetal malformation is discovered, the psychological effects increase [13]. Kaasen et al. reported on the psychological distress reactions after detection of all kinds of fetal malformations, but especially for the more profound malformations and for findings late in pregnancy [14]. Women in low-risk pregnancies have reported feelings of anger, despair and inadequacy at the time following a severe or lethal diagnosis [6]. Even physical symptoms such as sleeping or eating disorders are common [6, 15]. By going through a dynamic process, women can survive and cope with the traumatic event [15]. Lalor et al. describe this process as the stages *assume normal, being in shock, gaining meaning and rebuilding* [15]. The discovery of fetal malformations has the potential to create stress in the mother-to-be [6] and can adversely affect the mother-child relationship [5, 16]. The time between examinations is related to high levels of stress when vague findings are discovered [6]. Interactions with health professionals, getting time, space and structured counselling are of great importance in order to handle the situation [17].

Ultrasound examination plays an important role in diagnosing urogenital malformations, but it fails to detect all of the urogenital developmental [18]. One of the most common malformations is suspicious fetal HN, also called mild renal pyelectasis. It is most frequently noted in the left kidney and is more prevalent in males. A dilatation of the pelvis and/or calyces of the kidney are signs of suspected fetal HN. Consensus has yet to be reached regarding a measurement of the renal pelvis that is considered to be normal. However, the dilatation of renal pelvis is associated with risk for unfavourable outcomes [7]. It is difficult to accurately predict the outcome with dilatation between 4 and 10 mm. The dilatation can progress, regress or remain stable during gestation [7]. In Sweden, the follow-up ultrasound examination is typically scheduled between gestational week 32–34, about four months after the initial detection, and at this time dilation more than 7 mm is considered pathologic. HN does not generally cause any long-term problems if it is diagnosed and treated promptly. Undetected HN however could cause pain, either in the side and back or abdomen or groin. Other symptoms can include pain during urination or incomplete urination or incontinence, infections, nausea and fever. About 35 % of newborns with prenatal diagnosis of fetal HN show a normal anatomy upon ultrasound examination [19].

There are studies that relate to women’s experiences of prenatal diagnosis of major fetal malformation [6, 11, 20, 21], followed by decision of termination [11, 20, 22–24]. In the published literature we were unable to find research that focused specifically on women’s reactions to suspected HN.

The aim of this study was to explore women’s reactions to the discovery of fetal hydronephrosis in the context of uncertainty regarding the prognosis.

## Methods

Records of women who had given birth from 2009 onwards at a university clinic were examined in order to identify those who had been told that the fetus showed a significant dilatation of one or both kidney pelvises (dilatation of ≥ 5 mm) around pregnancy week 18–20. Most often, the detections had been made by midwives/sonographers and confirmed by an obstetrician within two weeks. A follow-up ultrasound examination was routinely scheduled by the obstetrician during gestational week 34.

The women were consecutively invited to an interview 6–12 months after delivery, by the obstetrician in charge (TG), and recruitment were based on the antenatal diagnosis. A total of 10 women consented to participate in the study. The mean age of the women were 30.6 years, six of them were nulliparous and four parous. TG and KS prepared the interview guide (Table 1). The semi-structured interviews were conducted by KS in a quiet room at the hospital, and each interview lasted about 60–90 minutes. The interviewer listened carefully to what was said, and the women were encouraged to speak freely. Follow-up questions were asked to give the women the opportunity to clarify relevant aspects of their initial answers. The interviews were recorded and transcribed verbatim by a secretary who had signed a binding agreement to secure confidentiality. Four women had college examinations and six university examinations. The women were given oral and written information, and they signed an informed consent before the interviews were conducted. Data collection and analysis took place simultaneously and continued until data failed to add any new information.

**Table 1:** Interview guide

What were your feelings and thoughts before the ultrasound examination?	
What was your experience of the ultrasound examination itself?	
How did you interpret the information given, and what feelings did this information bring out?	
What are your experiences of the first weeks after the ultrasound examination?	
Did the ultrasound examination finding affect your family or other persons around you? If so, how?	
What were your feelings just prior to the final ultrasound examination?	
What were your feelings after the final ultrasound exam?	
What were your feelings when your baby was born?	
With the experience you now have gained, do you think that the information provided before the delivery about the care that you would be given during your pregnancy, the delivery, and what you could expect post-partum should have been presented in some other way?	

Constant comparative analysis, which is the basis of Grounded Theory, was used to process data [25–27], i.e. this is not a Grounded Theory study but the method, constant comparative analysis from Grounded Theory was applied to the qualitative data. The transcribed interviews were analysed line by line according to Glaser’s scheme of open coding to generate substantive codes, i.e. words indicating a relationship with the research question [27, 28]. The codes were constantly compared to identify patterns as well as to generate categories [28]. Categories were also compared to each other and scrutinised to verify their relevance, and find out how they were related in order to identify a core category [28]. To improve the trustworthiness of our analysis, the data were reviewed several times by all authors. Disagreements were resolved through re-readings and discussions. The transcribed interviews were analysed by TG and KS, and MO reviewed the findings and all authors came to a consensus. Categories were confirmed by repeatedly returning to the original data and by ensuring transparency in the processing of data; all results can easily be traced to their origins.

The study was approved by the ethics committee of the university hospital, Linkoping, (the regional ethical board in Linkoping) Sweden (2010/127-31). Women were informed that they could decide not to participate or withdraw from participation at any time without giving an explanation and that their decision would not affect future healthcare encounters. They were also informed about where to get support if needed. The research team as well as an independent and experienced gynaecologist/therapist was available upon short notice for the women for counselling. This support mechanism was activated once.

## Results

The analysis resulted in four categories: 1.When the unexpected happens, 2. To live in suspense during pregnancy, 3. Difficulties in understanding information and 4. Suppress feelings and hope for the best. The core category, ‘Going through crisis by knowing that you are doing the right thing’ was identified, which explains the intersections of each category with the others.

### When the unexpected happens

This category is based on the thoughts assigned to two substantive codes: one code indicating those that were positive towards the screening and one indicating those that questioned the screening for HN.

Most women wanted to know as much as possible about the fetus, and a majority stated that they wanted to be informed if any fetal malformations had been identified. The most common emotion among the women in relation to the ultrasound examination was arousal and heightened interest. They were expecting to see a healthy fetus and to find out if it was a girl or a boy. When the ultrasound examination revealed a suspicious fetal malformation, most of the women were thankful that this had been found ‘in the abdomen’ and thought that it was better to have this information as early as possible, so they would have time to prepare themselves before delivery. To know early would help them to avoid total surprise or even severe shock in the delivery room.

*‘Of course, I felt sadness about being informed about hydronephroses, but, eh…, I am also a realistic person and I know there is a lot that can go wrong during a pregnancy. So, if there is something (with the coming baby), we want to know as soon as possible. I even tried to feel glad it wasn’t some fetal malformation in the brain or the heart’.*

The women put great trust in the examiners and thought they were very competent. After the detection of suspicious HN, the follow-up examinations were described as a safety net, that is, a standard part of the plan in which they were enrolled, and they did not question the need for follow-up. Although they had not been asked about their opinion, they described their own participation as positive and important.

*‘No, I never got that question (to withdraw from the examinations), it was like, I just got the information that we would do this check-up, and I never realised that I could say no, it was just taken for granted (laugh), and it was also a little exciting to see’.*

According to the women, the follow-up examination gave them a sense of security, since they knew what the next step would be. They hoped that the suspicious HN would disappear before the next examination, but they would have been satisfied if it had not develop into a harmful condition, and added that *‘if I was to be pregnant again, I would like to know the same way as this time’.*

Other women were more negative about the information given. Some women felt that the sonographer did not fully understand the women’s vulnerable situation at the screening procedure. The women came to the examination with positive expectations but also having their worst fear, which was that the examiner would find something seriously wrong. Most women had never heard of this condition, and the lack of knowledge created anxiety during the rest of the pregnancy. Some felt they did not need all the information they had been given, especially about all the complications that could follow, while others thought they got too little or confusing information.

*‘I would like to stress that I did not appreciate getting all the information so early in pregnancy. This information was given together with the assumption that the HN was not so big and probably not serious, and I felt like why do I need to know this now, and worry during half of the pregnancy if my baby probably is not affected by this?*

### To live in suspense during pregnancy

This category should be understood from the perspective that something wrong has unexpectedly been detected in the fetus causing a stressful situation.

Some women interpreted detection of suspected fetal HN as a sign that there might be more defects in the fetus. *‘Yes, I was terrified by the thought that the fetal malformation found could also mean that something more was wrong’*. Several women knew someone who had had this kind of experience. The worst fears expressed by the women were losing the child or delivering a child with multiple dysfunctions.

All but one woman said that they were worried during the rest of their pregnancy. Even if the diagnosis did not significantly affect their daily life, the worry, expressed as anxiety, nervousness, fear and stress was always present in the back of their minds.

*‘I did not go into any severe depression, I managed to do my daily work, and slept okay during nights, but all the time I had this background worries; there was always a feeling of worry in my head’.*

The women also said that the time they had to wait between examinations was a difficult time.

While they were waiting they had plenty of time to imagine worse case scenarios. Their worries related to whether or not they could trust the doctor and midwives and what the future would bring e.g. expressed as follows *‘maybe it will be severe fetal malformations after all and maybe something that has to be dealt with immediately after birth’*. Some women thought they could control their worries in between the examinations, but before the last exam and the delivery they got more and more worried and even had sleepless nights, wondering what would happen with their baby.

The women expressed ambiguity towards the ultrasound examinations. Examinations conducted by physicians were generally considered more stressful than the ones done by midwives, mainly because they did not really understand what was wrong with the fetus; some thought they got double messages like ‘*it looks fine but we will check again*’, which made them fear that “it” might be something much more serious than what the examiner wanted to say, maybe even fatal.

### Difficulties in understanding information

The knowledge about human anatomy and physiology varied widely in the group; some had no pre-existing knowledge at all while others had medical training and knew about HN even if they did not have experience with HN. Even if health professionals had said HN was rather common, no one in their families had heard about HN before and this contrast seemed odd to them.

Some women reported that they got insufficient information or explanation after the ultrasound examination. The information they received gave them no consolation. The information dealt with logistics and follow-ups. The actual measurements were rather abstract to most women, and the fact that the “problem” was measured in millimetres seemed to make it appear less serious. Some of the women said that they would not have understood anything without help from husbands or friends.

*‘No, we did not get so much information, mostly that it could lead to urinary tract infection, which needed to be checked directly after birth, but this was nothing to worry about now during pregnancy; the paediatricians will take care of it after birth. So, we really did not know much about what this was’.*

*‘She (the midwife) was measuring; however, I had difficulties to understand what she meant, but obviously something was wrong, and I got the impression it was not a serious fetal malformation, even if I did not know what 7 millimetres was related to’.*

Others appreciated that the health professionals took the time to explain thoroughly what they had seen on the screen because these explanations lessened or even took away their anxiety for a while. Only a few of the informants stated that they posed their own questions during the examination, and they felt that the ultrasound examination was not the best time to give medical information. One of the reasons was that they were so fulfilled and excited to see their baby at the ultrasound examination.

*‘No, I was so excited to see my entire child’s movement, so it was not easy to construct questions about the kidneys’. I would have wished to have a conversation in another room, sitting down at a table. It is difficult to discuss when you are lying down on an examination bed’.*

For the women, the most important questions were: What is HN? How great is the risk that it will not go away? What is the worst thing that can happen? To find answers to their questions and ease their anxiety, the women turned to friends or to the Internet, even if they had their doubts about the latter alternative. Their worst fear was that they would end up reading webpages with stories with sad endings that would only increase their anxiety. That happened to one of the women:

*‘No, I realised it was not about me, and I did not really understand the sentences, but I followed the discussion on the webpage, and one of the children died’*.

Despite this risk, on the whole, the women thought that information received on the Internet was useful. They also said that being able to follow other couples who were in the same situation was valuable. Friends and Internet were regarded as sources where one could address ‘stupid questions’ that the women dared not to bring up with the ultrasound examiner. Even those who thought that information given by the health professionals was enough stated that it was important to be able to find other sources of information.

*‘… but I think the Internet is excellent as an information source. Even if you have to think twice about all the information, you can at least get some more knowledge’.*

### Suppress feelings and hope for the best

Women expressed strategies to regain and maintain balance considering their new situation: supress their feelings and thoughts, look for more information and/or just try their best to be calm*.* The women stated that they did not understand how serious HN could be for their child. Either they interpreted the suspected HN as something harmless or experienced that the health professionals were very calm about it. Sometimes they thought that health professionals consciously played down the importance of what they found. Like the doctor saying that:

*‘He had to say that he saw very little change in the child’s kidney, which I interpreted as no danger. I started to ask a lot of questions, but my first thoughts were that I was glad all other things looked good, and thought that the world was not going to end with this finding’.*

A state of not being receptive helped the women to distance themselves from the fact that their child might get serious kidney problems in the future. Some told themselves that they would deal with “it” after delivery, *‘I did not permit myself to feel and think too much’.* Others stated that they did not allow themselves to be emotional and really sense their feelings at all. To deal with the time between examinations, many women found that devoting themselves completely to duties at busy workdays was a way to temporarily keep their anxieties at bay.

The women tried to reassure themselves that everything would be all right in the end. Health professionals as well as family helped them to keep up this strategy, and they described their surroundings as supportive and calming:

‘*I had the feeling that these check-ups were done as part of a routine, that it probably was going to be fine*’.

*‘My doctor told me it was not dangerous, and I also had a midwife telling me that of all severe fetal malformations that could be found at ultrasound examination, you are lucky it was not something else’.*

Another way of dealing with the anxiety, adapted to a relative perspective on HN, was also expressed by others:

*‘When they could tell me that nothing wrong was seen on the heart, and when they saw a little extra fluid in the kidneys it was like- well, at least it wasn’t in the heart’.*

### Core category *‘*Going through crisis by knowing that you are doing the right thing*’*

The core category answers the research question and usually describes a social process. In our case, ‘when the unexpected happens’ (category 1) represents positive feelings as well as questioning attitudes about the screening procedure. Our research question led us to investigate the questioning attitudes further. ‘To live in suspense during pregnancy’ (category 2) was described as the main situation and ‘difficulties in understanding information’ (category 3) meant that they sought information on their own. However, three different coping strategies could be described (category 4) as ‘suppress feelings and hope for the best’. Some women suppressed their feelings and thoughts, ‘*I did not permit myself to feel and think too much*’, and maybe realised that nothing could be done before delivery. Others looked for more information on the Internet sites or discussed with people in their surroundings: ‘*I want to find information and to get knowledge*’. Others tried to be calm and clung to the notion that ‘*it could have been worse*’. The core category ‘Going through crisis by knowing that you are doing the right thing’ shows the complexity that accompanies good intentions.

## Discussion

In Sweden, nowadays, prenatal screening and diagnosis are part of routine antenatal care and almost all women participate. The women in this study had positive attitudes towards the examination “and to see the child”, but the detection of suspicious fetal malformations created strong emotional reactions. The information caused feelings of anxiety and fear, which affected their pregnancy. The women handled the situation with different coping strategies such as suppressed their feelings, searched for information or distanced themselves and/or hoped for the best.

Ultrasound examination is a powerful method to improve pregnancy outcome, by providing detailed information about placenta localisation, gestational age and multiple pregnancies [29]. At the same time, it has also led to ethical dilemmas, for example, when major fetal malformations have been discovered: to terminate the pregnancy or continue [24]. This study shows that ethical dilemma also becomes difficult when a suspicious fetal malformation is detected. These parents received information about a detected malformation with unclear significance, and it was impossible for doctors and midwives to provide clarity and predict the outcome. Most of the parents reached a state of uncertainty which led to anxiety. One of the advantages with ultrasound examinations is that it seems to improve women’s connectedness and feelings towards the fetus [30, 31]. However, when major fetal malformations have been discovered, it creates stress [6] and initial reactions of shock [15] and may affect the mother-child relationship [5, 16]. It could also predict the quality of postnatal mother-infant relationship [32]. As the health of the fetus has been questioned for the women in this study, they might anxiously observe every deviation in their child’s development. They could perceive that the detected change in the ultrasound image is important, after all, for the child's development. Therefore, it is of great importance to communicate a realistic view of the discovered malformation and support the mother-infant relationship.

Ultrasound examination has the potential to create severe anxiety, especially when health professionals are not able to explain in a simple way [17, 33]. Even though women were given information, most of them complained about the lack of information, which is supported by previous findings [17, 34, 35]. In Sweden, midwives perform the routine ultrasound examinations but are not allowed to discuss adverse findings and diagnosis and should therefore contact a physician if fetal malformations are found. The women in our study considered that examinations made by physicians, compared to midwives, were more stressful probably because this could indicate a more severe situation. In order to minimise the situation when suspicious fetal malformations are detected, perhaps sonographers, irrespective of profession, could explain unexpected findings as Larson et al. suggest [33]. This requires that all healthcare professionals receive training so that they can meet both medical and psychological needs of women.

The women in this study stated that the examination room was not the appropriate place for a conversation about ultrasound findings. In an examination room, patients are in a vulnerable situation, as they are often undressed and in a dorsal position [36, 37]. Anxiety due to unfamiliar situation and facing possible danger with little knowledge or experience makes it difficult to absorb information [37]. Therefore, the women and their partners need to discuss and express their feelings in a comfortable environment. Setting aside time for counselling is also necessary. The standard time allotted for normal pregnancies is not sufficient, and organised follow-up care is essential for both suspicious and observed fetal malformations [6]. These actions may initially increase healthcare costs but in the long run, they will be cost-effective.

In this study, some women suppressed their feelings and/or distanced themselves while others sought more information. Individuals have different coping strategies when they are faced with stress [10, 38]. Women must be given the opportunity to express their anxiety, anger and be met with empathy [17]. At a later stage, they may be susceptible to more information and/or conversation. Others handle the situation differently and want information immediately. This is in line with Lalor et al. who recommend that health professionals must be attentive to every individual woman’s need for information [10]. Most women in this study had limited knowledge of anatomy and were not able to understand medical terminology. It is of great importance that the sonographer explains and helps the women to understand what is seen on the screen [30]. It would obviously be helpful to provide printed information with relevant and clear information as suggested by Lalor et al. [15]. Because the women felt that information was insufficient, many of them searched for information via the Internet. Nowadays, Internet is commonly used when information is needed, and online and offline resources are often compared [39]. However, more knowledge is needed concerning how the Internet is used by the parents. It is of great importance that health professionals have reliable Internet resources to recommend to these parents.

There was an obvious difference between the health professional’s routines and the women’s wishes concerning the need for follow-ups. The waiting period for the next ultrasound examination was experienced as chaotic and long, which is supported by earlier studies [17, 40]. It is evident that informing only once is not enough. Both women expecting children with major as well as suspicious fetal malformation are in need of continuing counselling and information [10]. The women are unprepared for negative information [8], and it is well-known that people in stressful situations use strategies to reject information [38]. The results of this study indicate that even a telephone call from a doctor or midwife sometime between exams could be very helpful. Additionally, opportunities to contact a psychologist could be discussed with the woman and her partner.

A limitation of the study is the retrospective design. However, we think it is important to understand the long-term effects on the mothers, following the discovery of an unclear finding during pregnancy, when maybe the life perspective has changed and the women retrospectively can discuss options in the caregiving they have experienced. It cannot be excluded that the richness of content would have increased if more interviews had been performed, but we considered the data as being sufficient to address the research problems. The results are still consistent and transferable to women in similar situations. Another limitation is also the lack of non-Swedish speaking and immigrant women. This study includes a homogeneous group of Swedish women, and further research needs to be carried out which includes women from different contexts, taking into consideration also the partner’s experiences.

## Conclusion

The medical purpose of ultrasound examination has been described as different from women’s point of view, and women are unprepared for adverse findings [6, 8]. Women are worried irrespective of suspicious or severe malformations. The women and their partners are confronted with something unexpected, and results from ultrasound examination are difficult to absorb. Regardless of suspicious or fetal malformations discovered, women should receive information and counselling tailored to their individual needs. The women must have the opportunity to express their feelings, ask questions and get feedback. Other sources of support could include: written information, links to reliable sources on the Internet and possibilities for repeated follow-ups.

## Abbreviations

HN: Hydronephrosis
